# Mothers More Altruistic than Fathers, but Only When Bearing Responsibility Alone: Evidence from Parental Choice Experiments in Tanzania

**DOI:** 10.1371/journal.pone.0099952

**Published:** 2014-06-25

**Authors:** Jana Vyrastekova, Janine Huisman, Idda Mosha, Jeroen Smits

**Affiliations:** 1 Radboud University, Institute for Management Research (IMR), Nijmegen Center for Economics, Nijmegen, The Netherlands; 2 Radboud University, Anthropology and Development Studies, Center for International Development Issues Nijmegen (CIDIN), Nijmegen, The Netherlands; 3 Muhimbili University of Health and Allied Sciences, School of Public Health and Social Sciences, Dar es Salaam, Tanzania; London School of Hygiene and Tropical Medicine, United Kingdom

## Abstract

Evolutionary theory predicts humans to be more altruistic towards genetically more closely related kin. Because fathers face uncertainty about the relation to their children, the asymmetric parental altruism hypothesis predicts mothers to provide a higher share of parental care than fathers. We tested this hypothesis using parental choice experiments in rural Tanzania, in which fathers and mothers could choose between an outcome that benefited themselves and an outcome that benefited their children. When a parent was solely responsible for the outcome, mothers chose more altruistic than fathers. However when the choice situation was changed into a coordination game in which responsibility was shared with the partner, the sex difference disappeared. Fathers then chose somewhat more altruistic, but mothers substantially less. Our findings thus partly support the asymmetric parental altruism hypothesis, but they also show that parental altruism is influenced by the context in which choices are taken.

## Introduction

The pattern of human parental investments deviates from that of other mammals in its exceptionally high paternal contribution [1]–[3]. This raises the question of how the parental investments are divided between fathers and mothers. Due to paternity uncertainty, the expected benefits from caring for offspring in terms of inclusive fitness are smaller for fathers than for mothers [4]. Also other facts of human reproductive biology, like high female initial investments into offspring (up to the pregnancy and lactation period) and low life-time fertility potential relative to males, might point towards sex asymmetry in parental investments [5]. These arguments can be summarized as the asymmetric parental altruism (APA) hypothesis, which proposes human parental investments by mothers to be higher than by fathers.

This hypothesis has received indirect empirical support from various angles. The extent of altruism between benefactors and beneficiaries has been found to depend on the degree of their relatedness [6]–[8]. Survival of children has been identified to depend predominantly on care by matrilineal kin [9], [10]. And, paternity uncertainty has been identified as predictor of paternal investments [11], [12]. The relevance of paternity uncertainty in the functioning of human societies was also found reflected in the widely spread sexual taboos [13], or in bans on promiscuity [14], [15] that can both be linked to paternity assurance. A controlled parent level test of the APA hypothesis is however not yet available.

The APA hypothesis plays not only a role in the evolutionary literature and anthropology, but has also been incorporated into models of economic decision-making, where it gained indirect support from studies on family consumption patterns [16]–[19]. This step represented an innovation of the traditional perspective in economics of the family as an atomic decision-making unit, in which parents were supposed to share the same altruistic preferences [20]–[23]. The question of whether mothers tend to invest more in their children than fathers is highly relevant for advising policy makers. When designing interventions for families living at the edge of poverty, it is important to know whether economic transfers should target the family as a whole or one specific parent [24].

In this paper we test the APA hypothesis by performing incentivized parental choice experiments in rural areas of Tanzania. To test for sex differences in egoistic versus altruistic parental decision making, we designed a one-parent treatment where unrelated fathers and mothers had to make a decision that could affect the welfare of their children. This treatment was designed to measure differences between mothers' and fathers' willingness to place their children's welfare above their own welfare, when holding full responsibility for such decision.

### Family context

Human parental decisions relevant for their offspring are often not taken in a vacuum but within the context of a family. From an evolutionary perspective, circumstances that supported the evolution of our large brain led us to excel in skills like speech and the formation and manipulation of social relationships [25]–[28]. It has been argued that these skills, in conjunction with other aspects of our evolutionary past – like intergroup competition and intragroup cohesion of kin-related males – are responsible for the emergence of stable parental bonds as an environment in which both parents invest into several offspring in a row. Consequently, the trade-off between parenting and mating in humans not only involves short term but also long term considerations [29]. The parental bonding in a family increases paternity certainty [30]; compensating fathers' fitness loss when foregoing external mating opportunities and investing into long-run parental care. Such advantages are likely to accrue in co-evolution with female preference for faithfulness [31].

The fact that parental decisions take place in the context of a family may have consequences for the symmetry of parental investments in their offspring. These consequences are however not addressed by parental altruism models that focus solely on the biology of human reproduction [32]. Family, as an environment which facilitates repeated interaction among parents, represents a mini-universe where parental decisions are likely to be shaped by mechanisms evolved in humans as social species [29], [30]. Next to testing the APA hypothesis, we therefore also test whether and how parental altruistic preferences interact with the context of human bi-parental care, taking place in a family.

To do so, we contrast two treatments. First, our one-parent treatment, where fathers and mothers had to choose between an outcome that benefited themselves and an outcome that benefited their children, without any involvement of their partner. Second, a both-parents treatment where the same decision was made by both parents of a couple, who knew that their partner was making a decision, but did not know what their partner chose. In the second treatment, the parents' beliefs and expectations about the behavior of the other parent - both in the experiment and after the experiment - may affect their behavior.

In the one-parent treatment, the situation is rather simple. Each parent has sole responsibility for the outcome and has to opt for the altruistic alternative if placing the welfare of a child over their own welfare. Furthermore, any parent can choose the selfish alternative without expecting blame or negative reaction from their partner for doing so. The decisions made by the parents in this treatment reveal their parental altruism under minimal social interference.

In the both-parent treatment, the situation is more complex, as the expectation of spousal approval or disapproval is now likely to affect the parent's choice as well. Moreover, both parents might prefer an outcome where at least one of them chooses the altruistic alternative, but at the same time, each parent might prefer that it is the other parent making the investment. This situation represents in game-theoretic terms a coordination problem, which can be modeled as the “game of chicken” or as the “battle of the sexes”, depending on whether the parents decisions are formulated in terms of the actions they choose (care/no care), or in terms of the sex-specific parental care norms (father cares/mother cares) [33].

The core of the problem is that each parent prefers the care to be provided by the other parent, while also the risk exists that no care is provided at all. Culturally determined sex roles may be important in this situation as well. Fathers might choose more egoistically than mothers, if they assume that it is a mother's task to care for the children. Mothers may choose more egoistically than fathers, if they consider it a father's task to provide for the family. Besides the short-run outcome, parents may also take potential future consequences of the decision into account. For example, expectations of negative reaction from their partner, after revealing no willingness to share responsibility, might lead them to choose the altruistic alternative. Our study of parental altruism in a family context is also in line with integrative approaches in evolutionary literature [34], [35], which call for considering a richer set of reproductive strategies when addressing differential parental investment behavior.

Decisions in human parental pairs are likely to depend on a range of factors that are absent when the parent is solely responsible for the outcome of the decision. To increase our understanding of the relevance of these factors, decisions of parents deciding alone will be compared with those made in the both-parent situation.

## Materials and Methods

The experiment took place in two neighboring regions in the North-Western part of Tanzania: Mwanza and Kagera. These are relatively small regions, representing 2.3% and 3.2% of the total land area of Tanzania mainland, respectively. Both are largely agricultural, with the exception of urban aggregations around a few cities. We selected six wards for our experiments, of which two in Mwanza and four in Kagera. Before starting the experiments, we ran test and training sessions in two other wards in the proximity of Mwanza city.

The participants in the experiment were recruited under the approval and with help of village leaders. Individual verbal consent was sought and obtained from study participants prior to their participation in the experimental games and survey interviews. We opted for verbal consent, because a substantial part of the people in the area of our study are illiterate and thus could not read the content of a written consent form. Because asking potential participants to sign a form they could not read would easily raise fear in them, we decided to use verbal consent in the local language to the local interviewers and to record the names of the consenting individuals in our logbook.

At each location, we performed two sessions; one in the morning and one in the afternoon. To prevent the spread of information from the morning session to the afternoon session, there was only a short break between the two sessions. The participants arrived at a central place in the ward, and were brought to a sheltered place (local school, church building), where the experiments could take place without intervention of non-participants. In all sessions we had the help of 10 trained assistants who were fluent in English and local language(s), and had previous experience with data collection in questionnaire studies.

At the start of a session, an assistant thanked to the participants in local language for their arrival. Then participants participated in another incentivized experiment, not discussed in this paper. This took about 45 minutes. The participants received no feedback about the outcome of this experiment before making the decision discussed in this paper. Between the events, there was a break of at least half an hour, involving time to relax and enjoy a small refreshment, and to answer a one-on-one questionnaire on demographic data. In this way, the two incentivized decisions were separated in time and context.

At the start of the session, we announced that the order of leaving the session would depend on the length of the questionnaire study, and that completing the questionnaire for men would take longer than for women. In that way, we created public knowledge that the women would leave the session before the men. We also asked the participants to leave the session location completely when they were dismissed and not to wait for other participants. In this way, we strived to guarantee that any decisions of parents, in particular mothers, would not be affected by the pressure of their partners waiting for them. The experimental group consisted of 188 parents (95 fathers and 93 mothers) with at least one child below the age of 10.

The game we implemented in the experiment was a modified dictator game. In the standard dictator game [36], the dictator is asked to divide a cash amount between him- or herself and a passive recipient. Transfers of the dictator to the recipient are interpreted as a measure of the dictator's altruism. In our study, parents were assigned the role of dictator and asked to make a decision affecting their own welfare as well as the welfare of one of their children. We used two treatments of this game. In the *one-parent treatment*, we invited only one parent from a family to participate; either the father or the mother. In the *both-parents treatment*, we invited both parents to participate. In the one-parent treatment the partners of the participants were not explicitly informed about the participation of their partner (although they might have heard about it after the experiment). In the both-parents treatment, the parents knew that their partner was participating in the choice experiment, but the partner was not present in the decision situation.

The choice task faced by the participants was as follows. We called the participants one by one to an isolated place, thanked them for their time and stated that as a reward, we would like to offer them the possibility to choose one out of two or three alternatives. In each of the sessions one of these alternatives was suitable exclusively as a consumption good for a child, namely a type of slippers worn by children in that area. The other alternative(s) were consumption good(s) that did not explicitly target the welfare of a child and could be consumed by the decision maker - an amount of cash and/or half a kilo of sugar (depending on availability). We also showed a bag with slippers in different (children's) sizes and colors, stating that they could choose slippers of another size or color than the one displayed. After the participant had made a choice and had left the building, the chosen alternative chosen was registered by the experimenters.

The slippers had a higher market value than the other non-cash alternative (sugar) and the cash amount. The purchase value of the slippers was about 1500 Tanzanian schilling, while the value of the sugar was about 1000 Tanzanian shilling, and the cash offered was between 500 and 800 Tanzanian shilling (depending on the banknote availability at the local bank). Choosing the slippers thus resulted in foregoing possible economic benefits for the parents themselves, in exchange for a more expensive good that can be consumed by (one of) their children.

We chose slippers as the child consumption good, because they considerably increase the welfare of children in the study area, who have to walk daily large distances, e.g. to go to school or for fetching water. The slippers are a valuable object. A large number of children in the areas of our experiment walked barefoot and half of our participants reported to have at least one child currently walking barefoot. Children with slippers often had old and worn ones. In order to prevent transfer of slippers to adults, the slippers from which the parents could choose were only available in sizes suitable for children, approximately aged two to ten. The participants were allowed to pick the desired slippers by size and color.

### Analysis

The data were analyzed using cross-tabulations and logistic regression analysis. We report Fisher's exact tests for contrasts between categories of subjects (per sex or per treatment). In the logistic regression models, the dependent variable was a binary variable indicating whether (1) or not (0) a parent would choose slippers instead of the selfish alternative (money or sugar). Independent variables were indicators for sex (mother versus father), treatment (one-parent versus both-parents), age of the parent and number of children under 10. The models also included fixed effects dummies to control for the (measured and unmeasured) differences among the six wards. To test for variation in sex differences between the one-parent and both-parents treatment, also the coefficient for the interaction between sex and treatment was added to the model. To obtain coefficients and standard errors for all theoretically relevant contrasts among the variables, several models with opposite coding of the sex, treatment and ward indicators [37] were estimated. For two women whose age was missing, average women's age in the treatment was substituted. Summary statistics of the data are presented in Table 1.

**Table 1:** Summary statistics.

Slippers chosen (%)	54,80%
Sex	
Female	49,5%
Male	50,5%
Treatment	
One-parent	49,5%
Both-parent	50,5%
Age, mean (SD)	37,4 (10,94)
Number of children, mean (SD)	2,5 (1,20)
Selfish alternative reward	
Sugar	23,4%
Cash	18,6%
Both sugar and cash	58,0%
Ward	
1	19,1%
2	17,6%
3	15,4%
4	11,2%
5	16,5%
6	20,2%
Number of participants	188

Prior to the study we obtained a research permit from the Commission for Science and Technology Tanzania (COSTECH) and our study, including the described consent procedure, was approved by and received ethical clearance from Muhimbili University of Health and Allied Sciences (MUHAS) Institutional Review Board in Dar-es-Salaam. We further obtained permission to conduct the study from the district, ward and village authorities.

### Data availability

The data reported in this paper are available from the corresponding author upon request.

## Results

**Figure 1:**
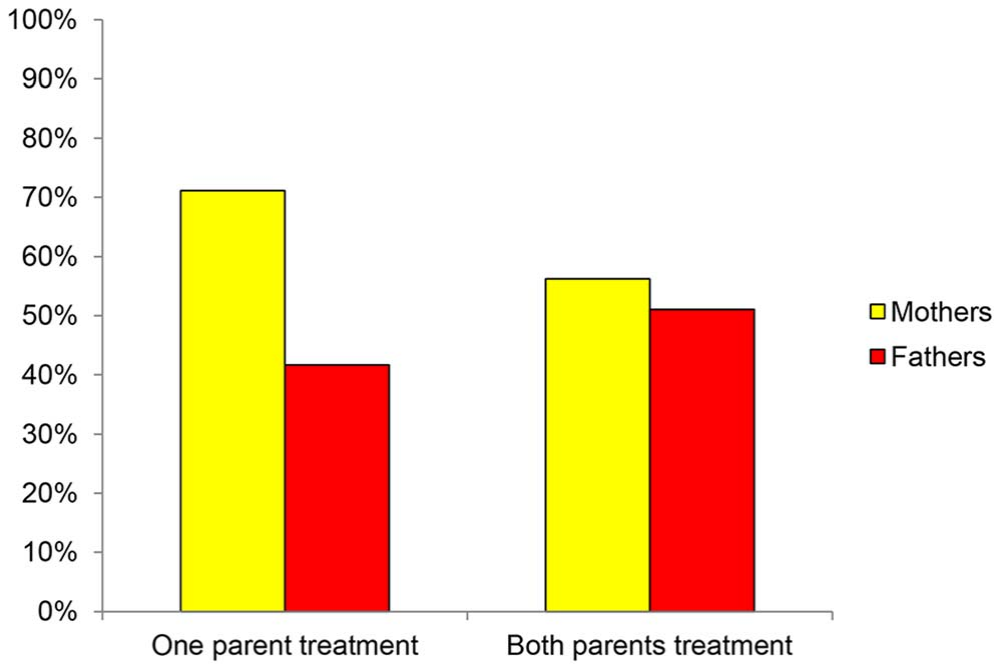
Percentage of altruistic choices by mothers and fathers per treatment.

For each parent, we observed the outcome of the choice between the altruistic and the more egoistic reward in the decision task. Bivariate analyses indicate that, on average, mothers were more altruistic than fathers; 63% of mothers and 46% of fathers were making the altruistic choice (Fisher's exact test, p = 0.020). This finding supports the asymmetric altruism hypothesis. However, this sex difference in parental altruism turned out to be only present in the one-parent treatment (where 71% of mothers versus 42% of fathers chooses altruistic, p = 0.006). In the both-parents treatment, the sex difference in altruism was not significant any more (56% of mothers versus 51% of fathers chooses altruistic; p = 0.683). Hence we only found support for the APA hypothesis in the one-parent treatment. These observations are summarized in Figure 1.

To find out whether the bivariate findings remain intact in a multivariate analysis, Table 2 presents the results of logistic regression analyses with the outcome of the parental choice (altruistic versus egoistic) as dependent variable. Independent variables are sex, treatment, parental age, number of children under ten, and indicator variables for the differences among the wards where the experiments took place. Two models were estimated. Model 1 contained only the main effects of the independent variables. Model 2 contained besides these main effects also an interaction coefficient, testing for the existence of sex differences between the treatment groups.

**Table 2:** Logistic regression models estimating the probability of the altruistic choice (child size slippers) by a parent.

	Model 1		Model 2	
	coeff.	p-value	coeff.	p-value
Sex (female vs. male)	0,726	0,040	0,742	0,038
Treatment (one-parent vs. both-parents)	0,248	0,449	0,283	0,395
Sex * Treatment			1,380	0,040
Number of children under 10	0,335	0,033	0,348	0,030
Age of the parent	−0,006	0,735	−0,007	0,694
Ward 1	0,383	0,271	0,354	0,315
Ward 2	−1,752	0,000	−1,853	0,000
Ward 3	1,067	0,008	1,110	0,006
Ward 4	0,049	0,904	0,100	0,809
Ward 5	−0,255	0,472	−0,222	0,537
Ward 6	0,508	0,130	0,512	0,131
N	188		188	
−2 Log likelihood	222,635		217,312	
Nagelkerke R square	0,240		0,265	

Model 1 makes clear that the significant sex difference in parental altruism observed in the bivariate analysis is also present when controlling for other important factors in the multivariate model. The significant positive effect of the sex variable shows that mothers choose significantly more for the slippers than fathers. This is in line with the APA hypothesis.

Regarding the other variables in the model, we observe that the coefficient for treatment is not significant. Hence on average there was no difference in altruism between parents who took the decision alone and parents who knew that their partner was making the same choice. As could be expected, parents with more children under ten significantly more often choose for the slippers. There were also some significant differences in altruism among the wards where the experiments were held, but the effect of parental age was not significant.

Model 2 shows that the coefficient of the interaction of sex with treatment is significantly positive. This means that the difference between fathers and mothers in altruism differs significantly between the one-parent and the two-parent session. To gain more insight into the pattern of these differences, we have computed coefficients for the theoretically most important comparisons on the basis of Model 2. These coefficients are presented in Table 3.

**Table 3:** Coefficients of theoretically most important comparisons based on Model 2.

Comparison		Coeff.	p-value
(1)	All mothers compared to all fathers	0,742	0,038
(2)	In one-parent treatment, mothers compared to fathers	1,439	0,004
(3)	In both-parents treatment, mothers compared to fathers	0,059	0,901
(4)	Fathers in both-parents treatment compared to one-parent treatment	0,399	0,380
(5)	Mothers in both-parents treatment compared to one-parent treatment	−0,981	0,046

In Comparison 1, we look at the effect of sex while pooling the data from the one-parent and both-parents treatment. Given the significant positive coefficient of this comparison, we are tempted to conclude that there are indeed sex differences in parental altruism, with mothers being significantly more altruistic than fathers. However, Table 3 also shows that this sex difference is only present in the one-parent treatment (Comparison 2) and not in the both-parents treatment (Comparison 3). This is in line with the findings of the bivariate analysis. When the participants know that their partner is making the same choice, the sex difference disappears (almost) completely.

What is happening here? Is it the fathers who in the both-parents treatment are more altruistic, the mothers who are less altruistic, or both groups moving to the middle? The coefficients of Comparisons 4 and 5 reveal the second option to be the case. Whereas the fathers seem somewhat more altruistic in the both-parents treatment, the mothers choose substantially and significantly less altruistic in that treatment compared to the one-parent treatment. Hence it is the mothers who mostly change their behavior when their partner is present and that change is in the egoistic direction.

## Discussion

We report data from parental choice experiments collected in rural Tanzania. Evolutionary biology argues that male uncertainty about parenthood implies less altruism towards offspring in fathers than in mothers. So far, only indirect evidence for this hypothesis was available. We provide new experimental evidence.

In our experiments we compare two treatments in which parents make a decision affecting the welfare of their children. In the first treatment, the parent making the decision bears sole responsibility for the outcome of the decision. This one-parent treatment allows us to test the asymmetric parental altruism hypothesis rather strictly, i.e. with little social interference. In the second treatment, both parents make the same decision simultaneously, though independent of each other. In this both-parents treatment, the parents are faced with a more complex coordination problem in which responsibility is shared. In this situation, not only the outcome for the children, but also the consequences for the parent in the parental pair might influence the decision.

In line with the APA hypothesis, we find that mothers are significantly more likely than fathers to trade off their own welfare for the welfare of their children. However, this asymmetry prevailed only in the one-parent treatment. If both partners participated, the difference between mothers and fathers disappeared. Moreover, the mechanism was driven by mothers decreasing their altruism in the both-parents treatment, relative to the one-parent treatment.

Hence, it seems that involvement of the partner decreased altruism among the mothers. This raises the question why this would be the case. As suggested in the introduction, it is possible that these mothers more easily made a selfish choice, because responsibility was shared and there was a chance that the partner would choose altruistically. Alternative explanations are that they considered it their husband's task to provide for the family, or that they expected their partner to choose selfish and did not want to be worse-off than him. The latter explanation of mothers' pessimistic expectations with respect to the partner's behavior is in line with cooperation of heterogeneous groups in Nairobi slums [38]. And, the slight increase in altruism of males suggests that the consequences of not providing parental care in a pair might play a role, although in this experiment clearly not a role of much importance. The decisions of fathers might also be affected by the certainty of parentage, which is unobservable in this study, or by incentives to signal own quality by providing parental care [35].

The validity of our findings might be affected by three important sources of bias. First, it is possible that slippers were chosen for their resale value instead of for use by the participant's children. Second, it is possible that the egoistic alternative (money or sugar) was in fact chosen on behalf of the children instead of for the parents themselves. And, third, it is possible that the observed differences between fathers and mothers are in fact more general differences between males and females.

The last option could not further be studied in our experimental setting, as our setup was specifically designed for use with parents. However, the other issues could be addressed to a certain extent by validity tests. In one of these tests, we asked a sample of parents without children under ten (28 males and 36 females) to make the same choice between slippers for young children and the more selfish alternative. It turned out that of these parents only 22% choose the more valuable slippers (against 55% of the parents with children under 10). This difference is statistically significant (Fisher's exact test, p = 0.000).

Whether the parents without children under 10 who choose the slippers did so for egoistic reasons (hence the resale value) is not clear. They may have chosen them also for other altruistic reasons, for example for their grandchildren. However the fact that these parents opted significantly less for the slippers than parents with young children supports the idea that the last group indeed chose the slippers for their children. This idea is further strengthened by anecdotic evidence obtained by observing the parents. They often went through a batch of slippers, comparing sizes and colors before making a choice, while smiling in the process of doing that.

The second possibility – that some seemingly egoistic choices could have had unobserved altruistic motives – cannot be ruled out completely. It is very well possible that in some cases parent choose cash to satisfy an urgent need of a child. However, such a situation would probably bias the outcomes towards lower identified altruism for mothers compared to fathers, as females in these areas tend to have less access to cash. Given that significantly less mothers than fathers opted for the egoistic alternative, unobserved altruistic motives do not seem to have played a role of importance.

Independent of the mechanism at play, the parental decision-making in a context of parental pairs was not in line with the APA hypothesis. This indicates that asymmetry in parental altruism may be overruled by factors driving parental behavior in a family context. Hence, interventions targeting the welfare of children are not only influenced by whether the mother or father takes the decision, but also by the circumstances in which the decision making takes place. When both parents are involved, the outcome may be less favorable for children than when mothers are solely responsible.
